# Supplementary material for: Mapping genes for human face shape: Exploration of univariate phenotyping strategies
Source: PLoS Comput Biol. 2024 Dec 2;20(12):e1012617. doi: 10.1371/journal.pcbi.1012617 (PMC11661606; doi:10.1371/journal.pcbi.1012617)

## S1 File

**Fig A. Boxplot of correlations between different groups of facial traits.** The pairwise linear correlation coefficients between each univariate phenotype from two groups of traits were computed using the 'corr' function in MATLAB R2023b. The median and standard deviation are displayed at the top, and phenotype categories are shown at the bottom of each subfigure.


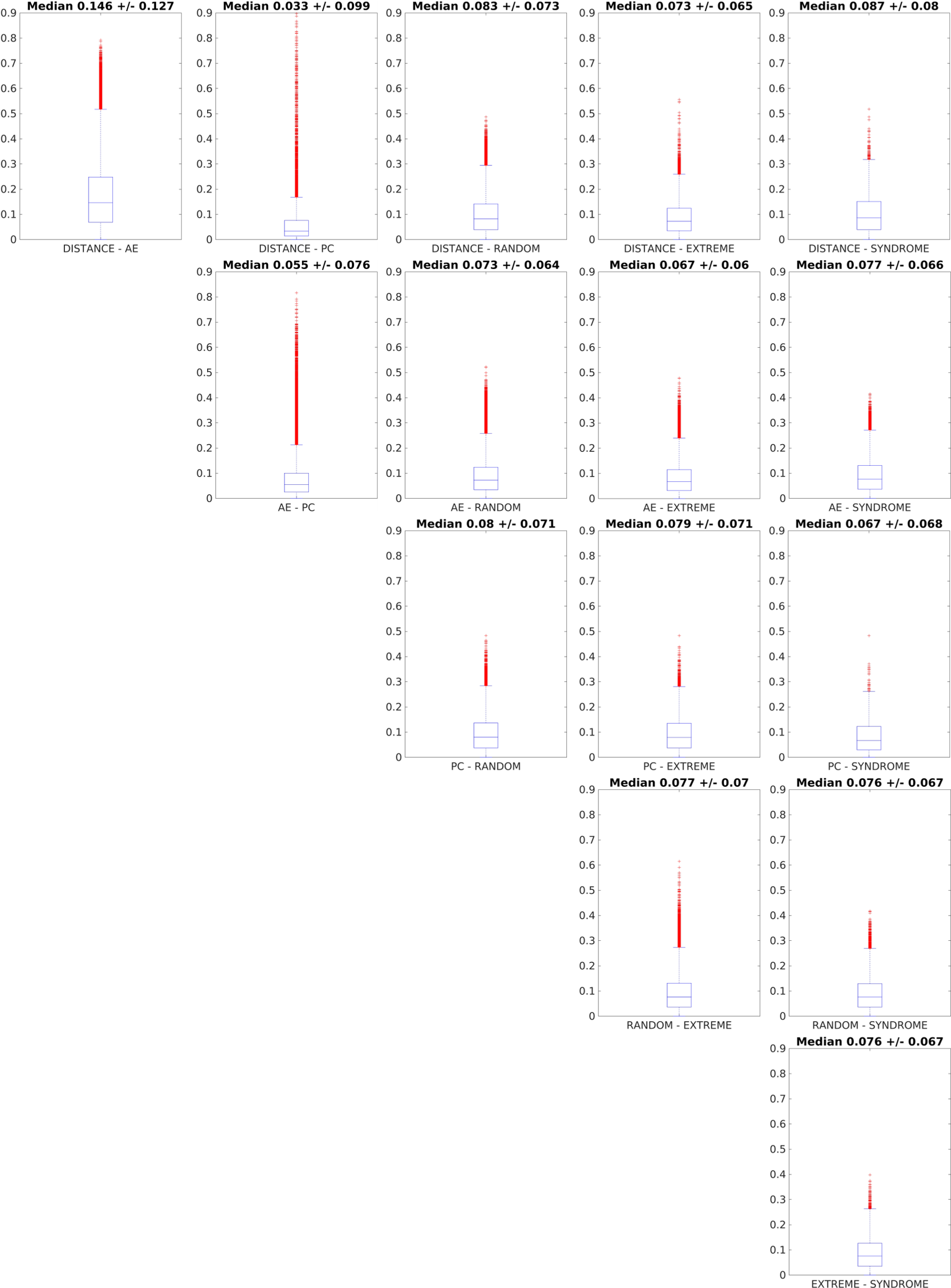


**Fig B. P-value matrix of pairwise differences in mean SNP-based heritability of different phenotyping categories.** Adjusted p-values, computed using the Benjamini-Hochberg procedure, from two-sample t-tests evaluating mean heritability differences among different categories of traits, including inter-landmark distances (DISTANCE), traits extracted by auto-encoder (AE), traits extracted by principal component analysis (PCA), resemblance scores to randomly selected examples (RANDOM), resemblance scores to extreme examples (EXTREME), and resemblance scores to syndromic examples (SYNDROME). Non-significant results (P < 0.05) are highlighted in red.


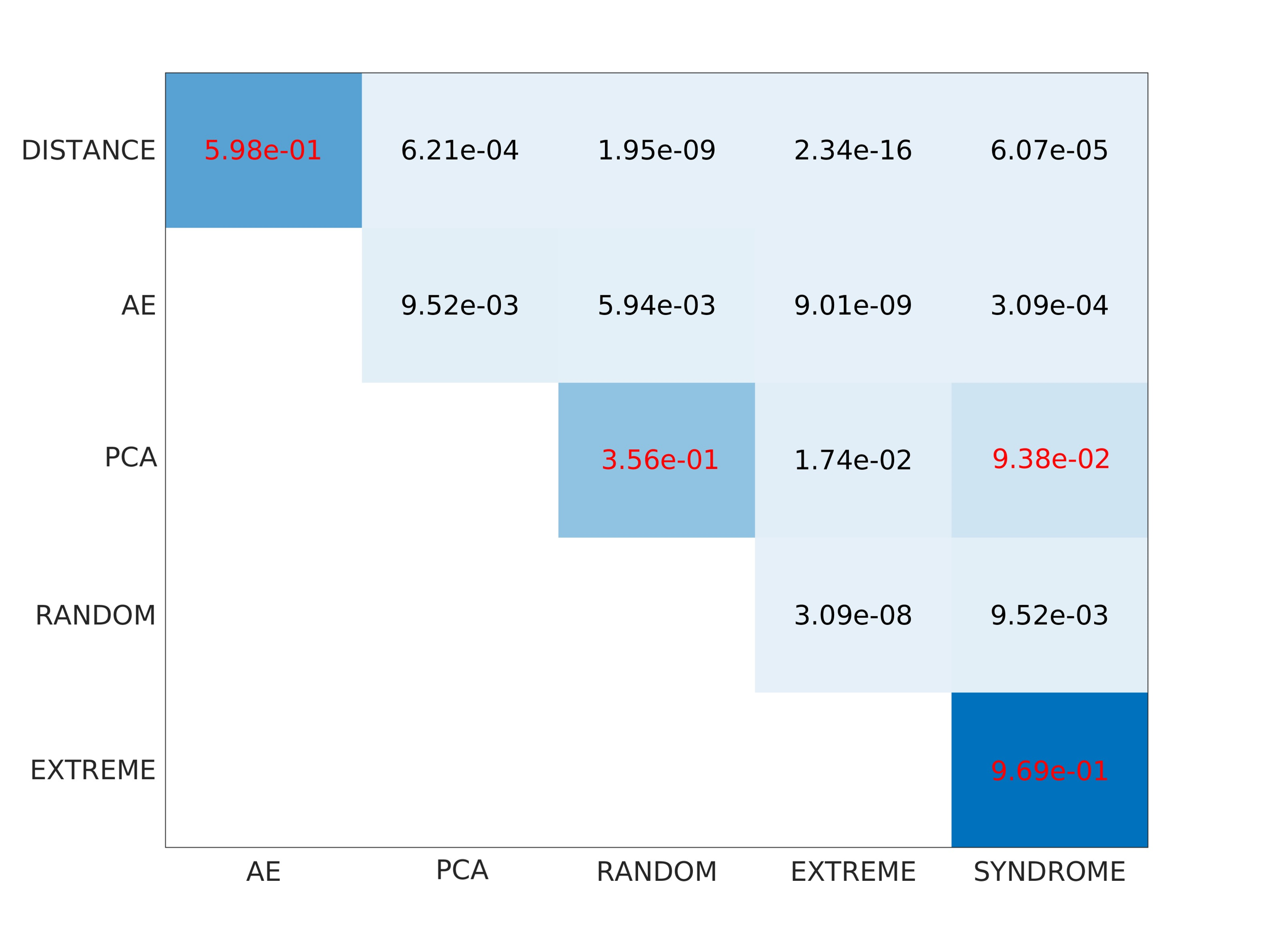


**Fig C.** **Comparison of SNP-based heritability between phenotyping categories for nasal and facial shape**. SNP-based heritability was estimated using LD-score regression. Facial (dark purple) and nasal (light purple) phenotyping categories include inter-landmark distances (DISTANCE), traits extracted by auto-encoder (AE), traits extracted by principal component analysis (PCA), resemblance scores to randomly selected examples (RANDOM), resemblance scores to extreme examples (EXTREME), and resemblance scores to syndromic examples (SYNDROME).


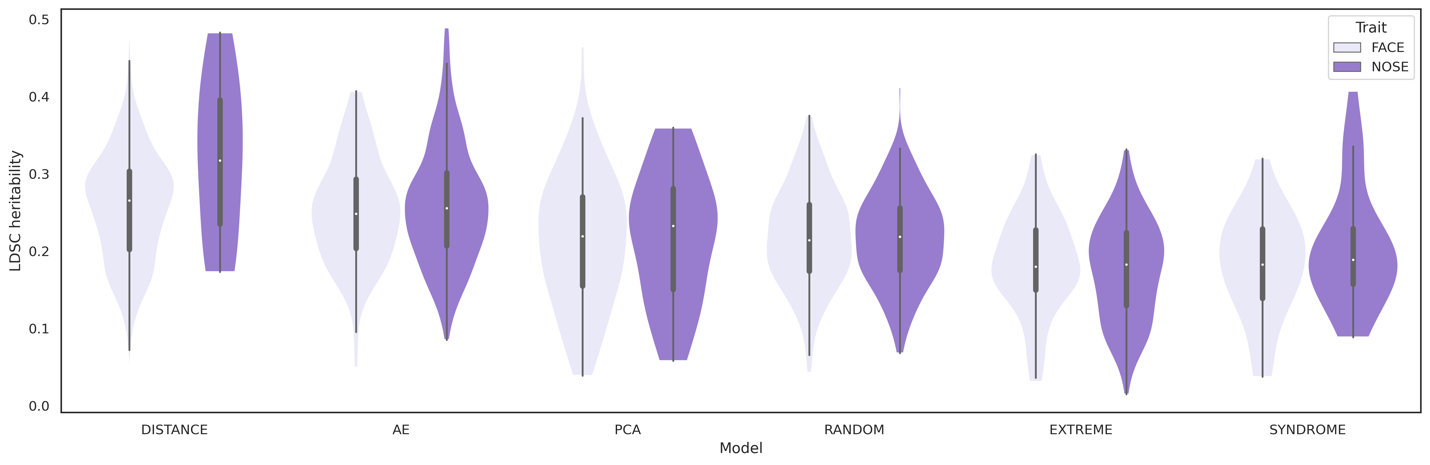


**Fig D. Comparing facial phenotyping categories in terms of independent genetic loci identified in GWAS.** The colors represent different categories of facial traits: green for inter-landmark distances (DISTANCE), dark blue for traits extracted by auto-encoder (AE), light blue for traits extracted by principal component analysis (PCA), light red for resemblance scores to randomly selected examples (RANDOM), medium red for resemblance scores to extreme examples (EXTREME), and dark red for resemblance scores to syndromic examples (SYNDROME). Unlike PCs, which are ordered according to descending explained variance, and resemblance scores to extreme gestalts based on the cosine distance to the mean shape, there is no specific order among other categories of traits. Therefore, given a fixed absolute number of traits, we randomly selected a subset 10 times from the full set of inter-landmark distances and resemblance to syndromic gestalts. Additionally, 10 replicates were performed for generating multiple AE latent dimensions and resemblance to random gestalts under different random initializations. The error bars represent the results from these 10 replicates.


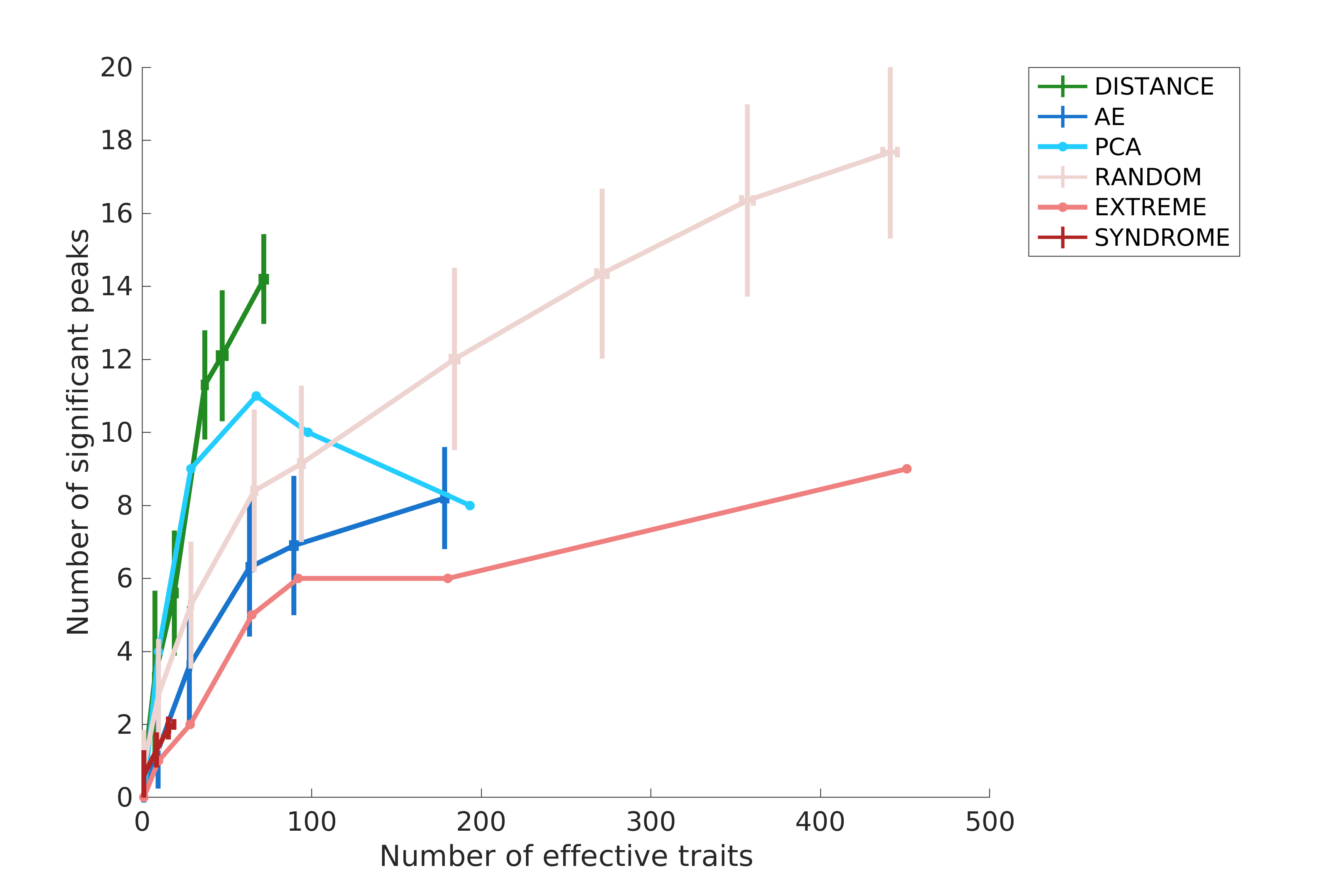


**Fig E. Frequency of genes identified by different categories of traits.** Phenotypes include inter-landmark distances (DISTANCE), traits extracted by auto-encoder (AE), traits extracted by principal component analysis (PCA), resemblance scores to randomly selected examples (RANDOM), resemblance scores to extreme examples (EXTREME), and resemblance scores to syndromic examples (SYNDROME). Due to random initializations, we included 10 replicates of AE dimensions and 10 replicates for resemblance scores to randomly selected gestalts.


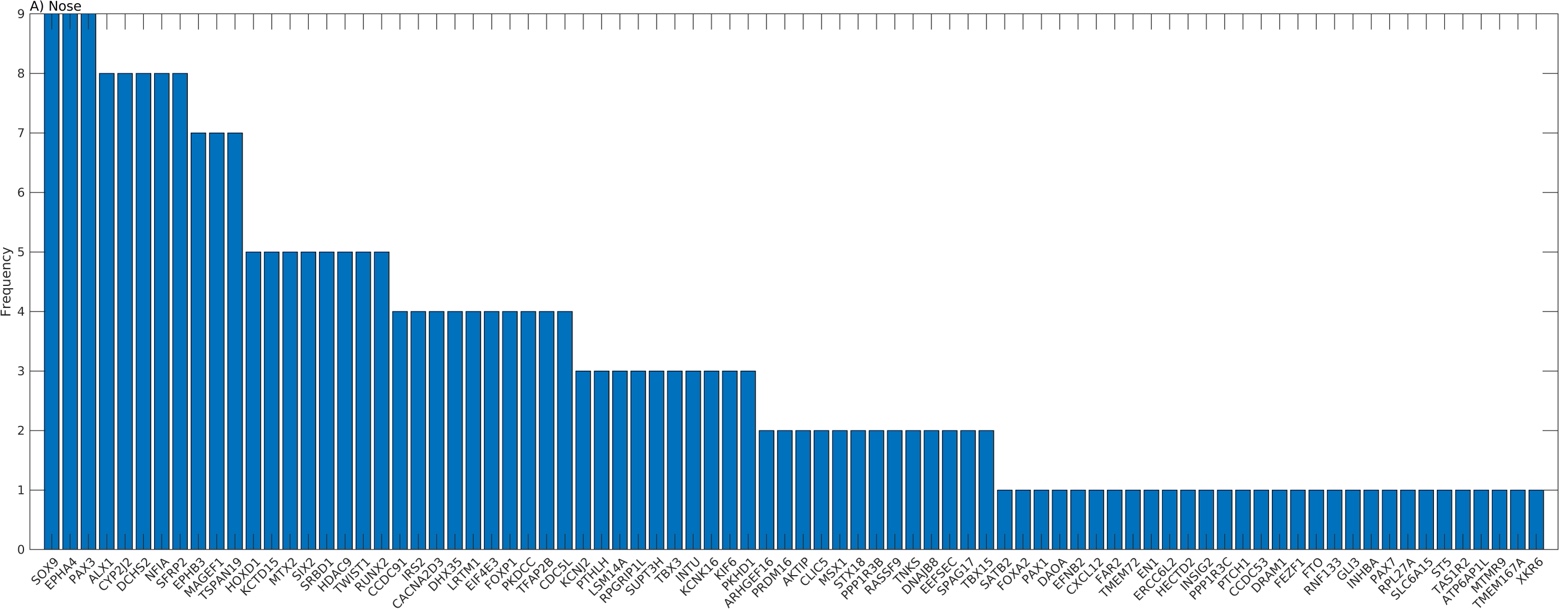


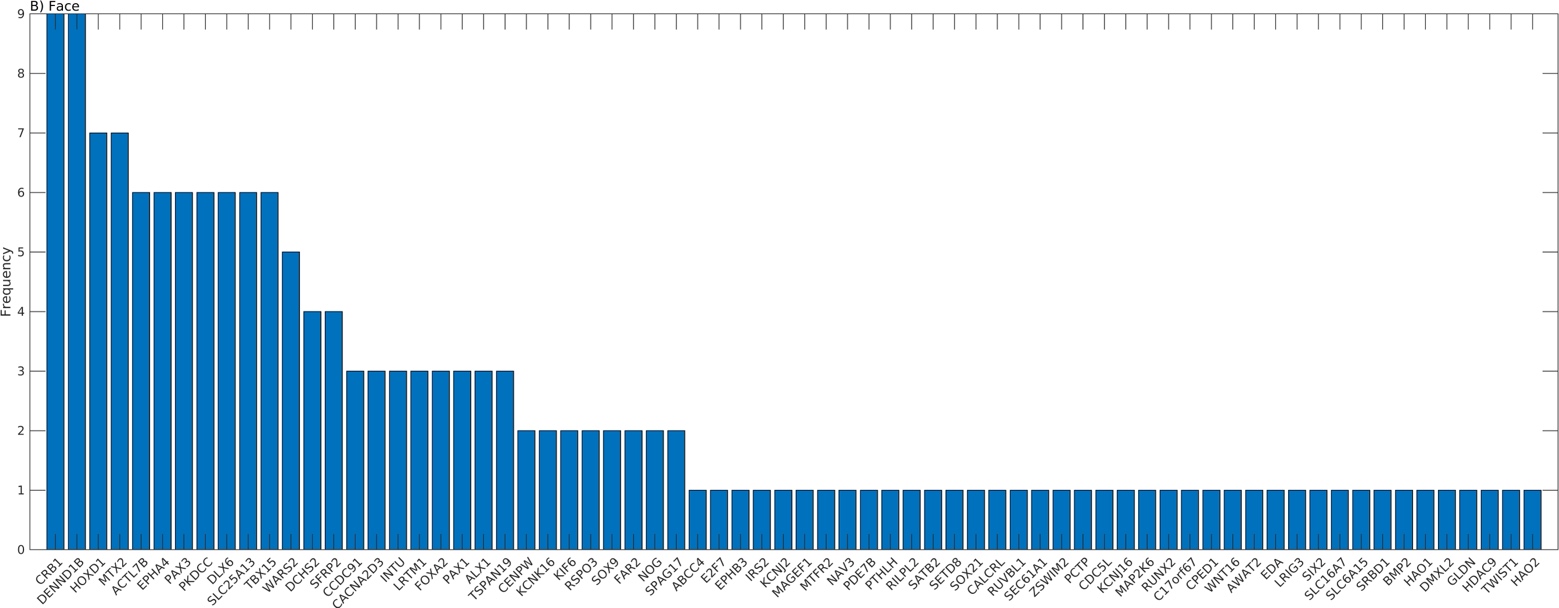


**Fig F. Using polynomial regression analyses to determine the optimal polynomial degree for each PC in predicting each dimension of AE.** Linear regression models (degree = 1) generally demonstrated best performance (lowest AIC).


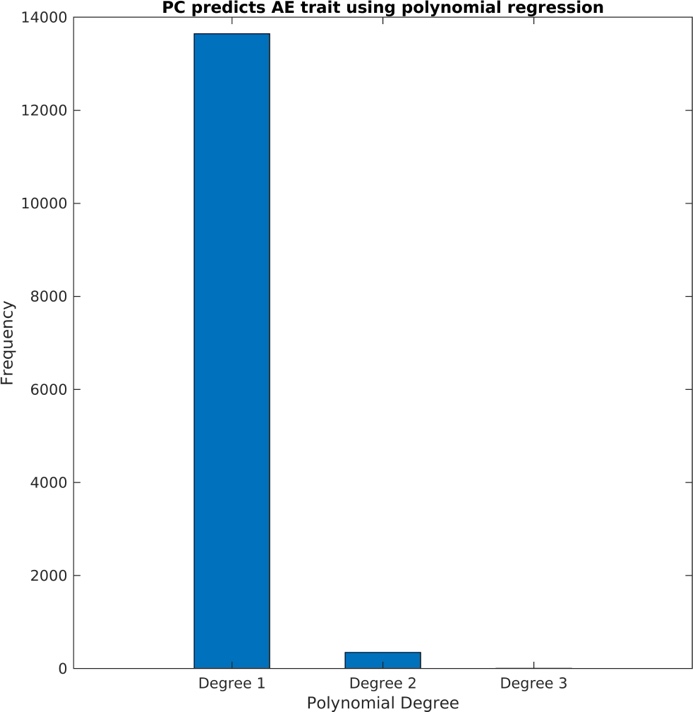


**Fig G. Locus zoom plot for SNP rs1999464 based on different phenotyping methods.**


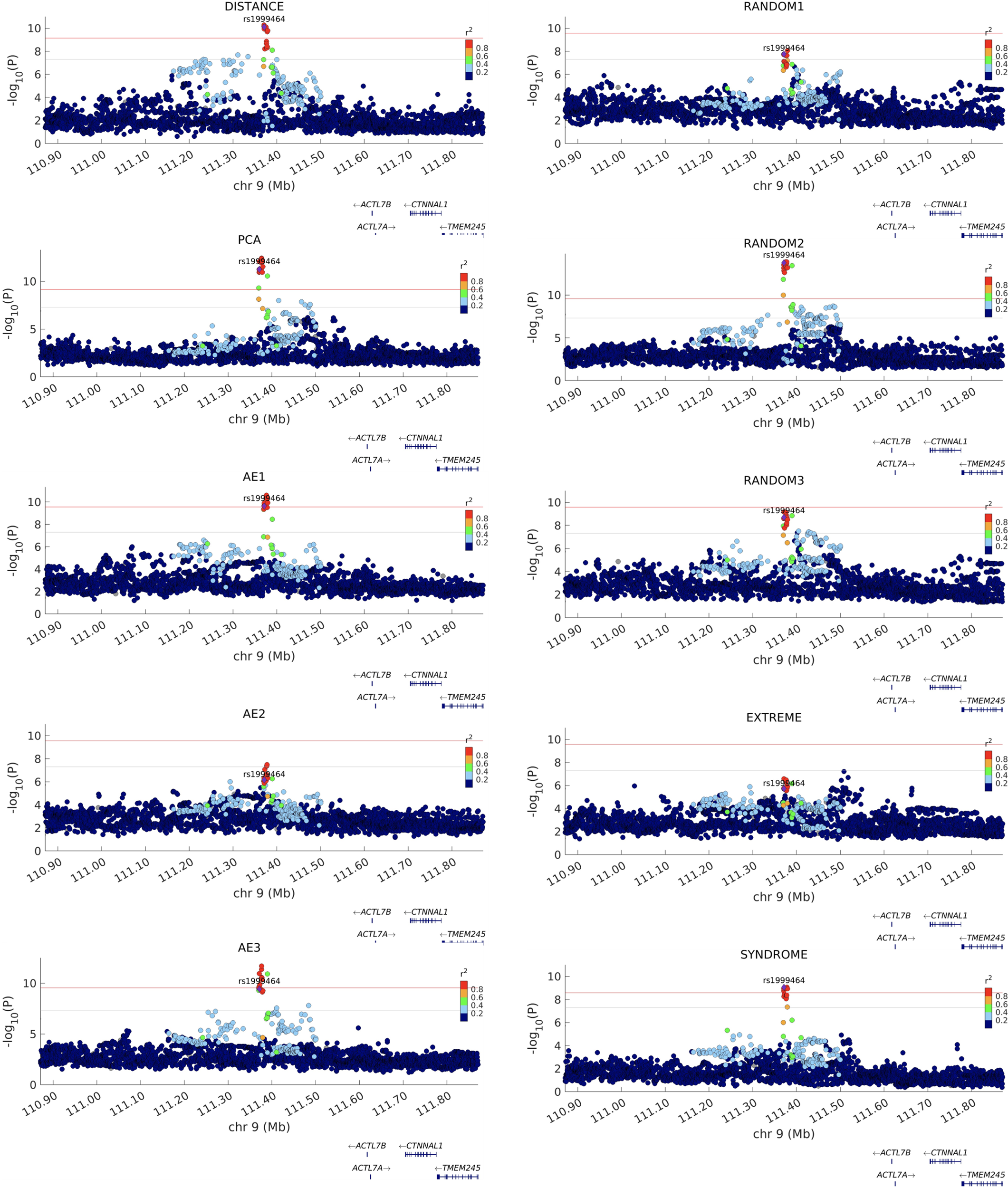

Supplement: S1 File — Fig A. Boxplot of correlations between different groups of facial traits. Fig B. P-value matrix of pairwise differences in mean SNP-based heritability of different phenotyping categories. Fig C. Comparison of SNP-based heritability between phenotyping categories for nasal and facial shape. Fig D. Comparing facial phenotyping categories in terms of independent genetic loci identified in GWAS. Fig E. Frequency of genes identified by different categories of traits. Fig F. Using polynomial regression analyses to determine the optimal polynomial degree for each PC in predicting each dimension of AE. Fig G. LocusZoom plot for SNP rs1999464 based on different phenotyping methods. (DOCX) [file pcbi.1012617.s001.docx]
