# Supplementary material for: Mapping genes for human face shape: Exploration of univariate phenotyping strategies
Source: PLoS Comput Biol. 2024 Dec 2;20(12):e1012617. doi: 10.1371/journal.pcbi.1012617 (PMC11661606; doi:10.1371/journal.pcbi.1012617)
Supplement: S1 Methods — (DOCX) [file pcbi.1012617.s003.docx]

## S1 Methods

Data were randomly split into a training (90%) and a test (10%) set for the construction and evaluation of the models. The AE network was trained for 200 epochs using the backpropagation algorithm [1]. The reconstruction error was defined as the mean absolute error (MAE) between the original shape and its reconstruction. To simplify the learning process, the data were first normalized by subtracting the average shape from all shapes. To prevent overfitting, we applied weight regularization [2] (weight-decay: 1e-6), encouraging a sparse weight matrix to reduce redundancy. We used the Adam optimizer [3] with an initial learning rate of 0.0001. To enable the optimizer to take smaller steps as training approaches convergence, we employed a learning rate scheduler, reducing the learning rate by 0.99 after every epoch. If not explicitly stated otherwise, default recommended values in PyTorch [4] were used for any other hyperparameters (amsgrad: False, betas: [0.9, 0.999], eps: 1e-08). To fit in the available GPU memory (11,264 MiB), we trained the networks in mini batches of 80 samples. The models were implemented and trained on an NVIDIA GeForce RTX 2080 Ti using PyTorch 1.9.

The comparable reconstruction error in the training and test sets shows that the model can correctly reconstruct both training samples and unseen test samples after compression.


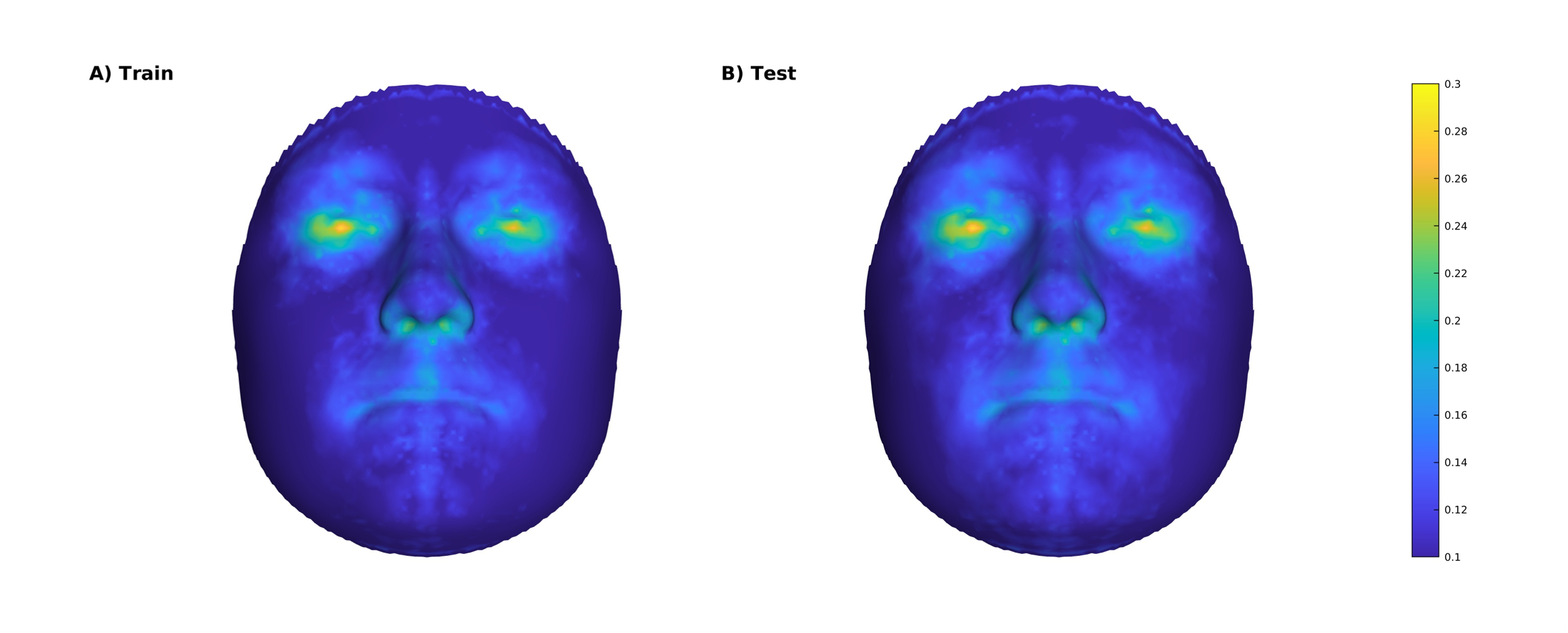


References

1. Kelley HJ. Gradient Theory of Optimal Flight Paths. ARS Journal 1960;30:947–54. https://doi.org/10.2514/8.5282.

2. Loshchilov I, Hutter F. Decoupled Weight Decay Regularization. ICLR, 2019.

3. Kingma D, Ba J. Adam: A Method for Stochastic Optimization. International Conference on Learning Representations 2014.

4. Paszke A, Gross S, Massa F, Lerer A, Bradbury Google J, Chanan G, et al. PyTorch: An Imperative Style, High-Performance Deep Learning Library. Adv Neural Inf Process Syst 2019;32:8026–37.
