# Supplementary material for: Mapping genes for human face shape: Exploration of univariate phenotyping strategies
Source: PLoS Comput Biol. 2024 Dec 2;20(12):e1012617. doi: 10.1371/journal.pcbi.1012617 (PMC11661606; doi:10.1371/journal.pcbi.1012617)
Supplement: Attachment — Submitted filename: ReviewerComments_PCOMPBIOL-D-24-00574.pdf [file pcbi.1012617.s004.pdf]

Title: Mapping genes for human face shape: exploration of univariate phenotyping strategies
Manuscript ID: PCOMPBIOL-D-24-00574

In this manuscript, the authors compared various phenotyping strategiest for assessing 3D facial shape in GWAS. The article is well-written, clear, and easy to follow. The content should be interesting to readers. While the methods looks reasonable, I have several concerns and comments:

1. In the abstract and results sections, the authors mention that the inter-landmark distances exhibit the highest SNP-based heritability as estimated by LDSC. However, Figure 3 shows that the number of genetic finding from GWAS based on the inter-landmark distances phenotypes is much smaller than other phenotyping categories. It is unclear that whether the genetic finding from other methods are not the false discoveries. I would suggest also showing the overall disease heritability for each phenotyping category. Higher heritability with fewer genetic findings may occur if the overall heritability of inter-landmark distances is higher than other categories. More discussions would be beneficial, and it would be helpful to show the overlaps across methods, not only show the pairwise overlaps as Figure 3, in VennPlot or UpSet Plot for different phenotyping categories including inter-landmark distances.

2. The authors utilized an AutoEncoder to derive latent variable from multiple phenotypes in the same phenotyping category for GWAS. While the AE approach appears to capture the similar heritability of the traits, however, the lack of a distinct training and testing split raises concerns about potential over-fitting. Over fitting could result in inflated associations that do not generalize to independent datasets.

3. The authors mention that 32 principal components (PCs) explain 99.21% of nasal shape variation, while 70 PCs explain 98.08% of facial shape variation. It is essential to provide an overall summary of the correlation across univariate phenotypes to understand the broader implications. Meanwhild, Figure 3A illustrates the effective number of traits for different numbers of traits selected. Correlations between PCs and autoencoders (AEs) may elucidate the non-linear relationships among phenotypes. This correlation analysis can shed light on how different phenotyping strategies capture varying aspects of genetic and structural complexity in facial morphology. Additionally, understanding these correlations can help in interpreting the genetic findings and their relevance to the phenotypic traits under study.

4. Minor comment: the authors state that no further adjustment for covariates was necessary as the facial surface scans were already adjusted prior to the calculation of any univariate phenotype in Lines 588-590. However, in GWAS, it is crucial to also adjust genotype data for covariates such as age, sex, weight, and height to account for potential confounding factors. I recommend the authors perform additional analyses to adjust for these covariates in the genotype data to ensure the robustness and accuracy of their GWAS results.
