# Supplementary material for: Mapping genes for human face shape: Exploration of univariate phenotyping strategies
Source: PLoS Comput Biol. 2024 Dec 2;20(12):e1012617. doi: 10.1371/journal.pcbi.1012617 (PMC11661606; doi:10.1371/journal.pcbi.1012617)
Supplement: Attachment — Submitted filename: Response.docx [file pcbi.1012617.s005.docx]

**Submission ID:** PCOMPBIOL-D-24-00574

**Title:** "Mapping genes for human face shape: exploration of univariate phenotyping strategies"

Dear Editor, and Reviewers,

On behalf of my co-authors, I would like to express our thanks for your comments and constructive suggestions. We have carefully considered them and revised the manuscript accordingly. In this letter, we give a detailed response to each comment. All changes made to the previously submitted manuscript have been highlighted in blue.

These revisions contributed to a significant improvement in our work. Therefore, we highly appreciate the efforts from the editorial board and reviewers, and we are looking forward to your reply.

Sincerely yours,

Meng Yuan

# Responses to Reviewer #1

Comment 1:

In the abstract and results sections, the authors mention that the inter-landmark distances exhibit the highest SNP-based heritability as estimated by LDSC. However, Figure 3 shows that the number of genetic finding from GWAS based on the inter-landmark distances phenotypes is much smaller than other phenotyping categories. It is unclear that whether the genetic finding from other methods are not the false discoveries. I would suggest also showing the overall disease heritability for each phenotyping category. Higher heritability with fewer genetic findings may occur if the overall heritability of inter-landmark distances is higher than other categories. More discussions would be beneficial, and it would be helpful to show the overlaps across methods, not only show the pairwise overlaps as Figure 3, in VennPlot or UpSet Plot for different phenotyping categories including inter-landmark distances.

Response:

Thank you for your suggestions. We agree that the high SNP-heritability of inter-landmark distances, despite fewer genetic findings, could benefit from further clarification. In the revised version of our manuscript, we have enhanced the explanation in several aspects:

- We have added a zoom plot for the initial intervals in Figure 3 to better illustrate our points in the discussion as follows. For nasal shape, where only five landmarks were available to extract pairwise distances, the number of identified independent genetic signals was limited. For facial shape, where more measurements were available, distances yielded a relatively high number of genetic loci compared to a similarly sized set of other traits. However, this comes at a computational cost, with the highest correlation observed among distances compared to other phenotyping categories. [discussion in Line 251-262].
- We have elaborated on the definition of the effective/independent number of traits to better illustrate the correlation among different measurements within one group of traits. [revised text in Line 152-156]
- The middle line of boxplot in Figure 2 shows the median SNP-based heritability for each phenotyping category. Additionally, we have provided descriptive statistics for heritability of different phenotyping categories in Supplementary Table S1.

Following these suggestion, we have replaced the heatmap in Figure 4 with an UpSet Plot to better visualize the overlaps across multiple phenotyping categories. Due to the extensive nature of the overlaps across 10 different models, we have included the comprehensive overlap information (the intersection of 3, 4, …, and up to 9 sets) in Supplementary Table S2-5 and Figure S5.

Comment 2:

The authors utilized an AutoEncoder to derive latent variable from multiple phenotypes in the same phenotyping category for GWAS. While the AE approach appears to capture the similar heritability of the traits, however, the lack of a distinct training and testing split raises concerns about potential over-fitting. Over fitting could result in inflated associations that do not generalize to independent datasets.

Response:

Thank you for highlighting this important point. We agree with your concern about potential over-fitting and appreciate the opportunity to clarify our approach. Although it was not clearly documented initially, we did implement a training/testing split in training AE. In the revised manuscript, we have added a detailed documentation on this procedure, including a comparison of reconstruction errors between the training and testing sets to demonstrate the generalizability of our model (in revised Supplementary File 2 - Implementation.docx). Both the AE and PCA showed similar performance in terms of accurately reconstructing the input shape after projecting it into latent space. For the subsequent genetic association analysis, the latent AE dimensions are treated in the same way PCA latent dimensions have been used in facial GWAS to date.

Comment 3:

The authors mention that 32 principal components (PCs) explain 99.21% of nasal shape variation, while 70 PCs explain 98.08% of facial shape variation. It is essential to provide an overall summary of the correlation across univariate phenotypes to understand the broader implications. Meanwhile, Figure 3A illustrates the effective number of traits for different numbers of traits selected. Correlations between PCs and autoencoders (AEs) may elucidate the non-linear relationships among phenotypes. This correlation analysis can shed light on how different phenotyping strategies capture varying aspects of genetic and structural complexity in facial morphology. Additionally, understanding these correlations can help in interpreting the genetic findings and their relevance to the phenotypic traits under study.

Response:

We appreciate the reviewer's suggestion to provide an overall summary of the correlation across univariate phenotypes. We have included boxplots displaying the distribution of pairwise correlations between phenotypes from different categories in Supplementary Figure S1. We observed that the median correlations are generally low, which is consistent with our findings that the extent of overlap in terms of genetic loci between different methods was relatively limited. [revised text in Line 386-388].

Furthermore, to explore potential non-linear relationships between PC and AE dimensions, we conducted polynomial regression analyses to assess the best-fit polynomial degree when using each PC to predict each AE dimension (Supplementary figure S7). Our findings indicated that linear regression models mostly perform best, suggesting that non-linearity is not prominently observed in static facial shapes. Although generally, non-linearity of data can be captured by AE models and may be insightful in various biological contexts, its added value is limited in the context of facial shape GWAS. [discussion in Line 311-322]

Comment 4:

Minor comment: the authors state that no further adjustment for covariates was necessary as the facial surface scans were already adjusted prior to the calculation of any univariate phenotype in Lines 588-590. However, in GWAS, it is crucial to also adjust genotype data for covariates such as age, sex, weight, and height to account for potential confounding factors. I recommend the authors perform additional analyses to adjust for these covariates in the genotype data to ensure the robustness and accuracy of their GWAS results.

Response:

Thank you for pointing this out. We agree that adjusting for covariates is crucial in GWAS to account for potential confounding factors. This was in fact done but was incorrectly written in the methods section. We apologize for the error. In this revised manuscript, we have corrected it. [revised text in Line 572-574]

# Responses to Reviewer #2

Comment 1:

Minimum P values was taken and then the Bonferroni method was applied to correct multiple testing across traits. This may be too stringent though Fig 3 suggest adding more traits still improved power. Authors may try to apply meta analysis to aggregate multiple traits within the same approach as mentioned in the introduction. Alternatively, the Sime's method may be more suitable for multiple testing correction across correlated traits.

Response:

Many GWAS meta-analyses, such as Fisher’s P, Stouffer’s Z, and inverse-variance weighted meta-analysis, assume that there is no sample overlap between GWASs. However, in our study, this assumption is invalid as there is complete sample overlap among all GWASs within each phenotype group. Consequently, we opted for an approach that does not depend on the assumption of non-overlapping samples, like the methods we used in [White et al., 2021, PMID: 33288918; Naqvi et al., 2021, PMID: 33821002]. Specifically, for each SNP, we selected the minimal P-value (minP) across all GWASs within each trait group. To address the issue of correlated traits, we estimated the number of effective independent tests per SNP based on the work of Kanai et al. (2016) PMID: 27305981. We conducted genotype-phenotype associations under the null hypothesis using permutation testing, which preserved the correlation structure of our phenotypes. This allowed us to adjust the genome-wide significance threshold (P < 5e-8) to a group-wide significance threshold by dividing P < 5e-8 by the effective number of traits, resulting in a method that is less stringent than the Bonferroni correction.

As suggested, we also applied Sime's method and compared it with our minP approach. In the Sime's method, firstly, the p-values are sorted in ascending order per SNP. Second, a meta p-value is calculated for each SNP using the formula $\min_{i} (P_{i}*\frac{m}{i})$, where m is the total number of phenotypes tested in a single category and i is the rank of the sorted p-value $P_{i}$. Using this formula, it can be derived that the most significant P-value receives a Bonferonni adjustment using the absolute number of traits, i.e., $P_{1,simes}=P_{1}*\frac{m}{1}$, whereas the minP approach we used, corrected this p-value using the effective number of traits, which is less stringent. While we understand that Sime’s method will provide a less stringent correction under certain conditions (due to the *min* operator), it did not in our case, where typically only one or a few traits showed a significant p-value. As illustrated in the figure below, Sime's method resulted in fewer significant peaks compared to the minP approach with permutation testing. This indicates Sime's method can be more conservative under certain conditions. More importantly, independently of the method used, the relative comparison between phenotype approaches does not change.

Comment 2:

Sharing of genomic signals were identified with a distance threshold of 250kb. This seems arbitrary. Please also show how many siginificant loci are overlapped without extending 250kb. Also it would be helpful to show the Manhattan plots from different methods in parrallel vertically so readers can check the sharing more closely. If possible, please try to use MASH (PMID: 30478440) to analyze the sharing of genetic effects between different phenotyping methods.

Response:

Thank you for your comments. The choice of a 250kb window originates from the default settings in FUMA (SNP2GENE) for determining independent loci based on GWAS summary statistics. Specifically, independent lead SNPs are defined if they are independent from each other at r2 < 0.1. Additionally, if LD blocks of independent significant SNPs are closely located to each other (< 250 kb based on the most right and left SNPs from each LD block), they are merged into one genomic locus. Each genomic locus can thus contain multiple independent significant SNPs and lead SNPs. [revised text in Line 219-220]

As suggested, we have included locus zoom plots for each significant SNP based on different phenotyping methods and arranged them vertically in parallel, enabling readers to more closely inspect the shared genomic signals. An example of a locus zoom plot can be found in Supplementary figure S8, with all figures available on figshare [https://doi.org/10.6084/m9.figshare.24867063.](https://doi.org/10.6084/m9.figshare.24867063.v2)

We appreciate your suggestion to use MASH to analyze the sharing of genetic effects between different phenotyping methods. However, we did not implement this method in our study due to specific requirements associated with MASH. This adaptive shrinkage-based method necessitates choosing a threshold and should ideally be applied to a full set of tests rather than only selected "significant" or "strong" ones. In our case, this would require a comprehensive SNP list and accommodate thousands of phenotype dimensions, which poses a computational challenge. Nonetheless, we acknowledge the potential value of MASH for future analyses and will consider it for subsequent research.

# Responses to Reviewer #3

Comment 1:

It is unclear how exactly many features were used in each analytic procedures. The numbers were scattered across manuscript and have to fish them out. Those numbers are critical, as the number of feature tested can have differential impact on the number of GWAS hits found. Larger the number, higher the chance to find high heritability and GWAS hits.

Response:

Thank you for your comment. We understand the importance of clearly presenting the number of traits used. To address this, we have updated the caption of Figure 3. The figure now includes the numbers in the caption, ensuring that readers can easily access this critical information without needing to refer to the methods section. Additionally, we have added a zoom plot for the initial intervals in Figure 3 to enhance readability. These changes could provide a clearer view for the impact of the effective number of traits on the identification of GWAS hits. [revised text in Line 649-654]

Comment 2:

It is very nice that the author attempted to address the issue of different number of tests involved in each different method, as shown in the Figure 3. But exactly because of the effective number of features are different across methods, the conclusion based on the distribution of the GWAS findings is weakened. As shown in the Figure 3B and 3C, the asymptotic behaviors have not been kicked in before the number of features are exhausted.

Response:

Thank you for your comment. That the asymptotic behaviours have not kicked in, is due to different reasons for different phenotyping strategies. However, we first want to point out that, thanks to using the effective number of traits and not the absolute number of traits, different phenotyping strategies can be compared in Figure 3B. For the inter-landmark distances, the number of distances were simply exhausted based on the number of landmarks used. For PCA, one can in fact observe a decline in discovery and hence the asymptotic behaviour has kicked in. This behaviour is expected for PCA, as it is well-established that later PCs primarily model noise in the data and are not expected to contribute to further genetic discoveries. For nasal shape the AE exhibits a similar pattern. For full facial shape, a similar pattern of initial increase and subsequent decline was observed for PCs. Here, we did not include more AE dimensions due to computational challenges. We generated multiple AE latent dimensions under different random initializations, conducting 10 replicates and subsequently 10 GWASs. This process is computationally intensive, for instance, 500 AE traits leads to 5000 GWASs. Typically, researchers determine the number of PCs based on an arbitrary percentage of variation explained. For example, a previous facial GWAS study [White et al., 2021, PMID: 33288918] retained 70 PCs to capture about 98% of the shape variation. In contrast, we conducted experiments up to 200 dimensions, which is already more extensive than other studies. Finally, for random and extreme gestalts, we conducted experiments with an absolute number of traits up to 500, as shown in Supplementary Figure S4. When expanding the number of random facial gestalts used to 500, this approach already outperformed all other methods. In other words, conclusions were possible, and additional experiments beyond those performed would not have added more insights.

Comment 3:

I found that the Fig4 is very confusing. It is hard to trace all the different numbers across a large matrices. It would be better if they can summarize the results with proportion instead of absolute counts. However, I acknowledge the difficulties in doing as, as the absolute raw count is already low.

Response:

Thank you for your comment. We understand that Figure 4 was confusing due to the difficulty in tracing different numbers across a large matrix. To address this, we have replaced the heatmap in Figure 4 with an UpSet Plot as suggested by reviewer 1. This visualization technique better illustrates the overlaps across different phenotyping categories. In the UpSet Plot, the total number of genes per phenotyping category is displayed when there is a single dot in the second panel. The corresponding number of overlapping genes with another phenotype group is also shown closely, providing readers with a clearer sense of the proportions. Additionally, we have included comprehensive overlap information, even for intersections of 3, 4, and up to 9 sets, in Supplementary Tables S2-5 and Figure S5. This supplementary data ensures that all details are available for those interested in the absolute counts as well as the proportions.

Comment 4:

The resemblence score approach is very interesting and has higher yield of GWAS hits despite low heritability. However, I am not sure what exactly those scores are trying to capture, especially those modeled with "extreme" cases and "syndrome" groups. It also makes me wonder what is exactly the number of effective features mean here, since there can be large number of "extreme" or "syndrome" groups that they have not modeled yet.

Response:

Thank you for raising this question. The idea of a resemblance score is to measure the presence of a specific facial pattern in an individual. These patterns can be defined in different ways, and the idea of syndrome [Vanneste et al., 2023, PMID: 38106188; Naqvi et al., 2023, PMID: 37024583] as well as extreme faces [Crouch et al., 2018, PMID: 29301965] as example patterns have been proposed in the literature. We agree with the reviewer that these are not the most obvious choices for GWAS, as indicated by their lower LDSC-heritability, and lower availability. Nevertheless, these are interesting to include to complete a broader view of what is possible.

We have expanded our discussion to provide more insight into the rationale for scoring traits along specific directions within the feature space (see revised text in lines 335-351). Additionally, we have included a visual representation demonstrating an example of a syndrome-driven trait in Figure 1.D.

It's important to note that the effective number of traits (as opposed to the absolute number of traits) is not the same thing as the number of effective traits. The effective number of traits refers to the number of independent phenotypes, determined through permutation testing (see Method in lines 586-597). This ensures equitable numerical comparisons across different groups of traits, focusing on statistical independence rather than implying clinical or biological significance.
