# Supplementary material for: Mapping genes for human face shape: Exploration of univariate phenotyping strategies
Source: PLoS Comput Biol. 2024 Dec 2;20(12):e1012617. doi: 10.1371/journal.pcbi.1012617 (PMC11661606; doi:10.1371/journal.pcbi.1012617)
Supplement: Attachment — Submitted filename: Response.docx [file pcbi.1012617.s006.docx]

**Submission ID:** PCOMPBIOL-D-24-00574

**Title:** "Mapping genes for human face shape: exploration of univariate phenotyping strategies"

Dear Editor, and Reviewers,

On behalf of my co-authors, I would like to express our thanks for your comments and constructive suggestions. We have carefully considered them and revised the manuscript accordingly. In this letter, we give a detailed response to each comment. All changes made to the previously submitted manuscript have been highlighted in blue.

Sincerely yours,

Meng Yuan

**Comment:**

In Figure 4 and Table S2-S5, the authors used the UpSet plot to represent the overlap of GWAS variants across different methods. It is good to classify them in terms of methods, however, this UpSet plot only showed the unique identifications and pairwise identifications. It would be beneficial to have an UpSet plot including all possible sharing patterns and also ranked by inclusive intersections. I understand the number of all possible sharing patterns should be a huge number, the authors could show the top 20-25 patterns. It could be more clear to show the relationship across different methods. Additionally, I have a follow up question about the consistency of the AE method. The authors replicated the AE method three times (AE1, AE2, AE3), but based on Table S2-S5, these three AE replicates identify different variants (overlap variants are few). It is better to have a discussion about the consistency of the AE method.

The reviewer addressed most of my comments. I have a minor suggestion regarding the current Figure 4, the UpSet plot. It would be better to categorize and rank the data into different phenotypic categories, similar to the organization in your previous Figure 4. For example, the top category could be DISTANCE, followed by the three AE replicates (AE1, AE2, AE3), and then the three RANDOM replicates (RANDOM1, RANDOM2, RANDOM3). This approach would help readers understand the similarities across the three AE replicates and the pairwise comparisons among them.

**Response:**

To provide a clearer representation of overlap across methods, we have updated Figure 4 to include as many sharing patterns as possible while maintaining readability. The patterns are first grouped by phenotype categories and then sorted by frequency, starting with all pairwise overlaps (21 combinations across 6 groups) and extending to the top 9 three-way overlaps, covering a total of 30 sharing patterns. Additionally, all possible overlap combinations, including up to six-way overlaps, are detailed in Supplementary Tables S2–S5 for further reference.

Regarding the consistency of the AE method and resemblance to random gestalts, we expanded our analysis to include both union and distinct sets of genetic loci across 10 replicates for each method (Supplementary File 3, Tables S6-S9). We then used the union set of loci from these 10 replicates to intersect with other trait groups. Additionally, we provided the highest and median intersection over union (IoU) rates to demonstrate the variability and stochastic nature of the results from these methods (revised text in lines 222-243, 404-408 and updated Figure 4).
